# Examining cellular responses to reconstituted antibody protein liquids

**DOI:** 10.1038/s41598-021-96375-8

**Published:** 2021-08-23

**Authors:** M. Tyler Nelson, Joseph M. Slocik, Eric J. Romer, Cassandra I. Mankus, Richard T. Agans, Rajesh R. Naik, Saber M. Hussain

**Affiliations:** 1 711th Human Performance Wing, Airman Systems Directorate, Air Force Research Laboratory, Wright-Patterson Air Force Base, OH 45433 USA; 2 Materials and Manufacturing Directorate, Air Force Research Laboratory, Wright-Patterson Air Force Base, OH 45433 USA; 3 UES Inc., Dayton, OH 45433 USA; 4 Oak Ridge Institute for Science and Education, Oak Ridge, TN USA; 5 The Henry M. Jackson Foundation, Bethesda, MD 20817 USA

**Keywords:** Biochemistry, Biological techniques, Biotechnology, Cell biology, Chemical biology, Molecular biology, Engineering, Materials science, Nanoscience and technology

## Abstract

Protein ionic liquids (PIL) are a new class of biologic stabilizers designed to protect the functionality and extend the shelf-life of biotechnological and therapeutic agents making them more readily available, and resistant to austere environments. Protein biorecognition elements such as monoclonal antibodies are commonly utilized therapeutics that require the robust stabilization offered by PILs, but biocompatibility remains an important issue. This study has focused on characterizing the biocompatibility of an antibody based PIL by exposing multiple cells types to a cationized immunoglobulin suspended in an anionic liquid (IgG-IL). The IgG-IL caused no significant alterations in cellular health for all three cell types with treatments < 12.5 µg/mL. Concentrations ≥ 12.5 µg/mL resulted in significant necrotic cell death in A549 and HaCaT cells, and caspase associated cell death in HepG2 cells. In addition, all cells displayed evidence of oxidative stress and IL-8 induction in response to IgG-IL exposures. Therapeutic Ig can be utilized with a wide dose range that extends into concentrations we have found to exhibit cytotoxicity raising a toxicity concern and a need for more extensive understanding of the biocompatibility of IgG-ILs.

## Introduction

Protein Ionic Liquids (PILs) are composed of cationized proteins electronically neutralized with a stoichiometric amount of anionic polymers that exhibit unique stabilizing properties in the water-free state^1^. These water-free protein ionic liquid formulations are designed to protect the structure and function of protein-based biologicals from destabilizing conditions such as oxidation, elevated temperatures, and pH fluctuations that can arise during storage. Furthermore, many PILs exhibit increased miscibility in both organic and aqueous solvents; and as a result, require reconstitution and dilution in buffer for use because of high protein concentrations (~ 750 mg/mL) and poor mass transport. PILs have been developed to stabilize a variety of proteins including ferritins, lysozyme, glucose oxidase, lipases, myoglobin, and a plant virus^2–4^. This study is focused on examining the biocompatibility of immunoglobulin (e.g. antibody) ionic liquids (IgG-ILs) after reconstitution in buffer, as antibodies represent the most prominent form of biorecognition elements that have revolutionized diagnosis and treatment of disease, research technologies, and monitoring health and performance. PIL stabilization of clinically utilized antibodies could significantly enhance cost savings and efficacy by extending the half-life and sustaining the stability of the functional elements of these products.

Numerous studies aimed at benchmarking the safety of ionic liquids (and some PILs) resulted in a body of literature demonstrating a broad spectrum of toxicities that range from non-toxic to very toxic depending on the specific chemistry of the IL^1^. A study characterizing cationized Ig (cIg) found that while treatment of HL60 cells with 10–1000 ng/mL cIg enhanced cellular uptake, it did not impact viability. It is important to note that this form of Ig was not anionically stabilized in the form of an IL and was characterized at a clinically low concentration range^5^. The wide array of reactive ionic liquid formulations coupled with the complex biochemistry of immunoglobulins could result in compounds with unanticipated cellular interactions and biodynamics leading to toxicity. Subsequently, since IgG-ILs could be utilized in a multitude of biological contexts, preclinical characterization of the cytotoxic effects of IgG-ILs reconstituted in buffer is warranted and can inform the selection of the most appropriate IL formulation for IgG-ILs utilized with biological tissue. To date, there is no published data examining the cytotoxicity of IgG-ILs reconstituted in buffer, thus compelling the design of this study to survey the effects of IgG-IL exposure on a variety of epitheliod cell lines (A549/lung, HepG2/liver, and HaCaT/epidermis) derived from potential target tissues. We examined the impact of µg/mL IgG-ILs on cellular viability, reactive oxygen levels and stress cytokine production. While the IgG and anionic component alone did not elicit a significant toxicological response in the cells, IgG-IL exposure at concentrations in excess of 12.5 µg/mL elicited dose dependent cytotoxic effects that appear to be cell type dependent.

## Results

### Material characterization

**Figure 1:**
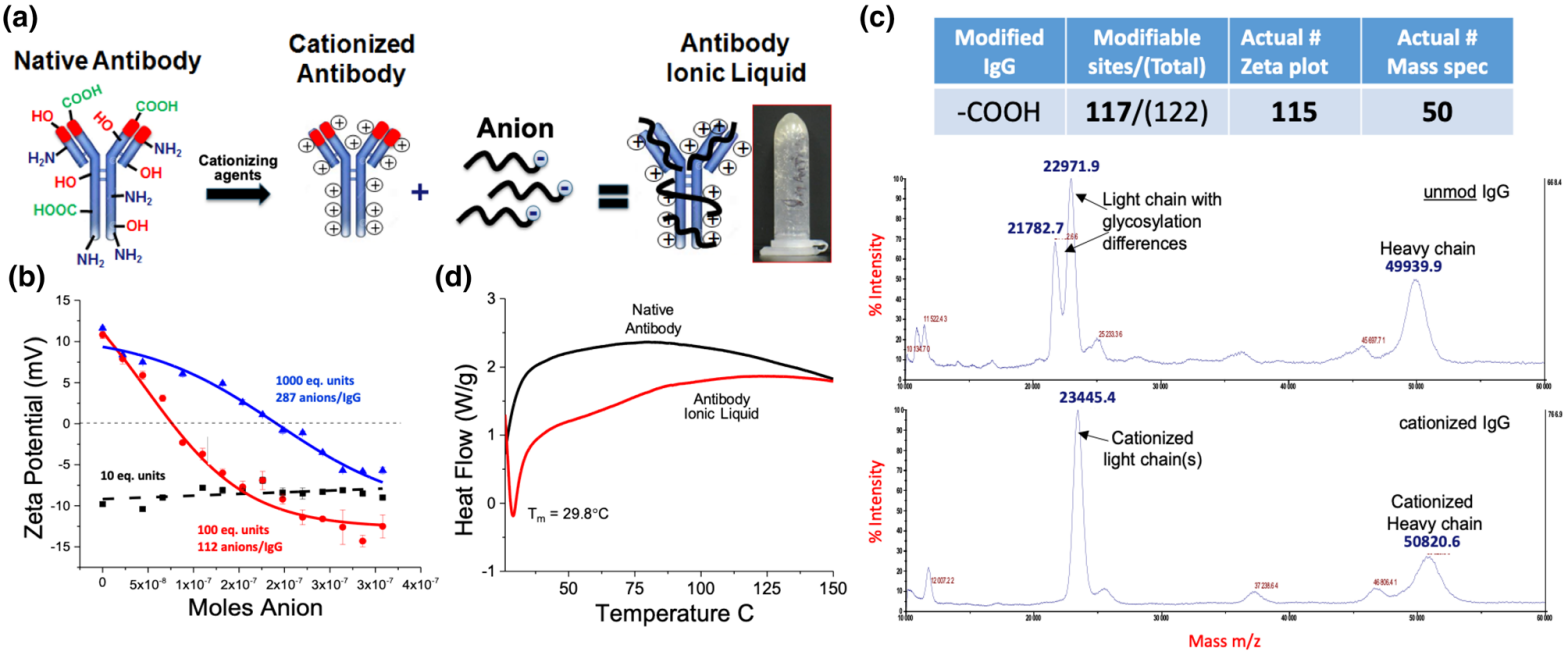
(**a**) Graphical representation of the method to produce antibody ionic liquid (IgG-IL). (**b**) Zeta potential charge analysis showing the differential impact of the free anion alone (10 equivalent units = 10 eq. units), 100 eq. units of free anion and cationized IgG (formulation utilized in the biocompatibility experiments described within this manuscript), and 1000 eq. units of the IgG-IL with respect to number of free anions added in solution. (**c**) MALDI mass spectrometry and (**d**) differential thermal analysis (DTA) material characterization IgG-IL (100 eq. units of free anion) highlighting the absorbance shifts in the peak intensities for the unmodified IgG antibody and the cationized form.

IgG-ILs were prepared by cationizing IgG antibodies by addition of positively charged groups of 3-dimethylaminopropyl 1-amine (DMPDA) and electrostatically paired with a stoichiometric amount of the anionic Poly(ethylene glycol)4-nonylphenyl 3-sulfopropyl ether potassium salt to create a viscous water-free antibody ionic liquid (Fig. 1a). For cationization, we used an EDC coupling reaction (1-ethyl-3-(3-dimethylaminopropyl)carbodiimide) to covalently modify the accessible carboxyl containing amino acids on the surface of Anti-IgG with multiple positive charges. This created a uniform distribution of surface charged sites around the antibody for complexation with anions. In total, we empirically modified the antibody with 10, 100, or 1000 stoichiometric equivalents of positively charged DMPDA and then experimentally determined the actual number of positive charges that were successfully added after extensive purification using zeta potential measurements. From the titration plots of zeta potential, we introduced 112 positive charges per antibody for the 100 equivalent stoichiometry and 287 groups per antibody for 1000 equivalents (Fig. 1b). The addition of 112 positive charges is in agreement with the 100 equivalents of DMPDA used for coupling and confirms the coupling reaction between carboxyl and DMPDA was highly efficient. At 100 equivalents, we partially modified the total number of available carboxylate groups per antibody. Theoretically, an IgG molecule composed of 2 light chains and 2 heavy chains contains ~ 144 acidic amino acids/1600 total amino acids based on a general IgG sequence. Furthermore, addition of tenfold excess DMPDA (1000 equivalents) ensured the likely modification of all carboxyl containing amino acids present within the antibody molecule; however, resulted in substantially more positive charges (287) than the theoretical number of ~ 144 carboxyl containing amino acids per immunoglobulin (IgG) antibody. At a high excess of coupling reagent, the EDC reaction likely loses specificity for carboxyl groups and broadly reacts with many other functional groups (ie. sulfhydryl and hydroxyl) to generate additional modifications. The 10:1 stoichiometry resulted in a negligible modification to the overall negative charge on the antibody based on zeta potential measurements. In parallel, we also characterized the cationized antibody by MALDI mass spectrometry to determine the number of positive charges introduced on the antibody surface. The MALDI mass spectra showed an increase in m/z for both the light chain and heavy chain of the cationized antibody vs the native and unmodified antibody (Fig. 1c). This increase in mass confirmed the addition of 50 positively charged surface groups of DMPDA per antibody. After the stoichiometric addition of anions and subsequent lyophilization, we determined the solid–liquid transition temperature of the cationized antibody/anion pair using differential thermal analysis (DTA). By DTA, the unmodified native antibody showed a small broad exotherm from 25 to 150 °C, indicating denaturation; while the antibody ionic liquid exhibited a sharp endothermic peak at 29.8 °C due to melting and a phase transition (Fig. 1d).

We measured antigen binding of antibody ionic liquids to determine the effects of ionic liquid formation (i.e. cationization and complexation with anions) on the biorecognition function of the Ig. To test binding, an anti-horse spleen ferritin IgG-IL liquid was incubated with an apoferritin target via an immunoblotting assay (Supplementary Figure 1)^6^. Briefly, the immunoblotting format involved immobilizing apoferritin on a nitrocellulose membrane, blocking membrane with BSA, incubating with reconstituted Anti-horse spleen ferritin ionic liquid sample, washing, incubating with a goat Anti-rabbit secondary antibody conjugated with alkaline phosphatase, and developing immunoblot with colorimetric substrate. As a result, the immunoblot confirmed that antigen binding of Anti-horse spleen ferritin antibodies was not affected by converting antibodies into IgG-ILs as observed by detection of apoferritin at low pM concentrations; however, more rigorous binding, thermal stability, and shelf-life studies are ongoing^6^.

### Biocompatibility analysis

**Figure 2:**
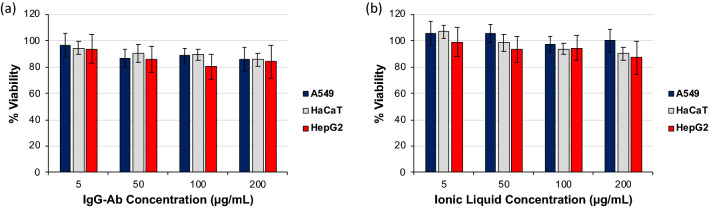
Viability results for A549, HaCaT, and HepG2 cells exposed to (**a**) IgG antibody or the (**b**) unconjugated ionic liquid solution for 24 h. Results were normalized to the no treatment condition for each cell line, and a one-way ANOVA, with a post-hoc Tukey’s analysis was conducted to determine statistical significance. * denotes p-value < 0.05.

A comprehensive toxicological approach was employed to evaluate cytotoxic effects of IgG-ILs at early (8 h) and later time points (24/48 h). We utilized multiple assays to evaluate cellular viability (LDH, dye exclusion) and death (caspase 3/7 activity). Fluxes in reactive oxygen species commonly observed with exposures to charged species was measured with an oxidizable probe (H_2_DCFDA). Control Ig and IgG-IL treatment concentrations utilized in this study spanned the low to mid µg/mL range to model therapeutically relevant dosing with Ig^7^. We first examined cellular viability/death following exposure to cationized IgG antibody and free anion solution to determine if the individual components of IgG-IL elicited cytotoxic effects (Fig. 2, Supplementary Figures 2–4). No statistically significant decreases in viability were observed for control IgG-antibody of free anion solution treatments at any concentration for all cell line.

#### A549-lung alveolar cell line

**Figure 3:**
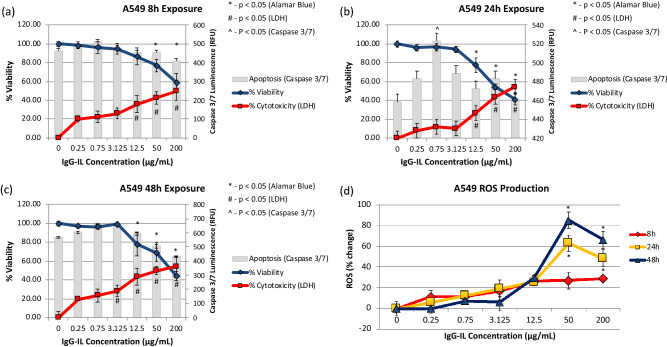
The viability (blue line, left y-axis), cytotoxicity (red line, left y-axis), and apoptosis (grey columns, right y-axis) results for A549 cells exposed to IgG-IL for 8 (**a**), 24 (**b**), or 48 (**c**) hours. Reactive oxygen species generation at 8, 24, and 48 h (**d**). Statistical significance was determined by a One-way ANOVA and a post-hoc Tukey’s Test compared to no treatment, at a confidence of 95% for n = 3, r = 6.

IgG-IL treatment of A549 cells resulted in minimal changes in viability/death after 8 h with lower IgG-IL concentrations (< 12.5 µg/mL), but higher concentrations (≥ 12.5 µg/mL) generally decreased viability (Fig. 3a). Similar but enhanced trends in decreased viability were observed at 24 (Fig. 3b) and 48-h following exposure (Fig. 3c) to higher IgG-IL concentrations (≥ 12.5 µg/mL), as was evidenced with the additional 20% decrease in viability by the 24 h time point. Caspase 3/7 activity was not significantly increased in any of the treatments, except the 0.75 µg/mL treatment measured at 24 h, suggesting the mechanism of cell death did not involve caspase 3/7 mediated apoptosis. By 8 h, ROS levels were close to control for all exposures (Fig. 3d). The 50 µg/mL treatment induced a time dependent increase in ROS levels relative to control at 24 and 48 h. There was a slight reduction with the 200 µg/mL treatment relative to the 50 µg/mL treatment at 48 h which likely represents probe leakage from dying cells.

#### HaCaT-keratinocyte cell line

**Figure 4:**
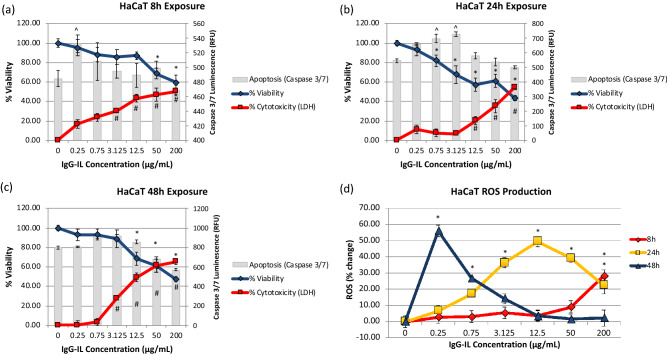
The viability (blue line, left y-axis), cytotoxicity (red line, left y-axis), and apoptosis (grey columns, right y-axis) results for HaCaT cells exposed to IgG-IL for 8 (**a**), 24 (**b**), or 48 (**c**) hours. Reactive oxygen species generation at 8, 24, and 48 h (**d**). Statistical significance was determined by a One-way ANOVA and a post-hoc Tukey’s Test compared to no treatment, at a confidence of 95% for n = 3, r = 6.

Viability decreased in a concentration dependent manner by 8 h post IgG-IL treatment with LDH showing significant decreases at lower concentrations (≥ 3.125 µg/mL) than the dye exclusion assessment (≥ 50 µg/mL) (Fig. 4a). Those general trends in decreased viability were sustained or enhanced at 24 and 48 h. For instance, the peak loss in dye exclusion viability (~ 60% decrease) was observed at 24 and 48 h with the 200 µg/mL concentration (Fig. 4b,c). Only modest increases in caspase activity were observed at lower concentrations that did not exhibit a consistent and sustained decreased in viability (Fig. 4a–c), suggesting again that IgG-IL cytotoxicity is not acting through caspase 3/7. Figure 4d displays a much different ROS production profile for HaCaT skin cells as compared to A549 cells. ROS production for HaCaT cells was negligible for 8 h treatment up to 50 µg/mL, whereas at 24 h, statistically significant increases in ROS were observed at 0.25 µg/mL, peaking at a 48% increase compared to the control with the 12.5 µg/mL treatment. 48 h exposures resulted in significant ROS production for low (0.25, 0.75, and 3.125 µg/mL) concentrations, and negligible ROS production at higher concentrations, which may indicate dye leakage from dying cells due to the concomitant increase in LDH activity.

#### HepG2-liver cell line

**Figure 5:**
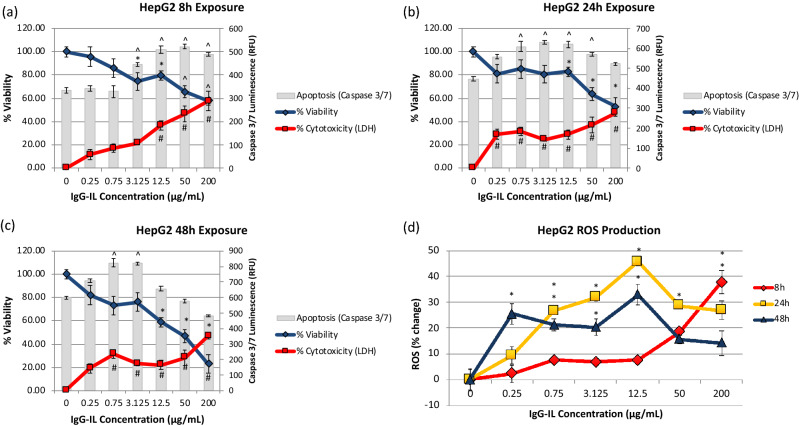
The viability (blue line, left y-axis), cytotoxicity (red line, left y-axis), and apoptosis (grey columns, right y-axis) results for HepG2 cells exposed to IgG-IL for 8 (**a**), 24 (**b**), or 48 (**c**) hours. Reactive oxygen species generation at 8, 24, and 48 h (**d**). Statistical significance was determined by a One-way ANOVA and a post-hoc Tukey’s Test compared to no treatment, at a confidence of 95% for n = 3, r = 6.

Higher concentrations of IgG-IL (≥ 12.5 µg/mL) induced a significant decrease in both measures of cell viability by 8 h (Fig. 5a). These decreases were generally sustained at 24 h and progressed to further declines in viability by 48 h, culminating in an 80% loss in viability with the 200 µg/mL treatment by 48 h (Fig. 5b,c). Interestingly, treatments as low as 0.75 µg/mL significantly increased LDH activity by 24 h and the result was sustained at 48 h. Caspase 3/7 activity was significantly increased with ≥ 3.125 µg/mL treatments by 8 h. This profile of increased caspase activity shifted by 24 h to include the 0.75–50 µg/mL treatments, and by 48 h only the 0.75–3.125 µg/mL were significantly different from control (Fig. 5a–c). This early induction of caspase activity is suggestive of an early apoptotic response, and correlates with the decreased viability and LDH cytotoxicity measurements. Future studies will be required to determine and characterize mechanisms of Ig-IL-mediated apoptotic cell death in this cell line. The progressive decrease in caspase 3/7 activity at the most cytotoxic concentrations may indicate a shift from late apoptosis to necrotic cell death. HepG2 cells increased ROS with IgG-IL treatments over time, with only the highest-level treatments significantly increasing ROS by 8 h and all but the lowest level treatment exhibited peak levels of ROS by 24 h. Interestingly, there was a general depression of ROS levels at 48 h relative to 24 h, with the exception of the lowest level treatment (0.25 µg/mL) that exhibited a pronounced significant increase in ROS (Fig. 5d). Together these results suggest the HepG2 cell line is sensitive to IgG-IL treatment, which can induce ROS production and cell death involving caspase 3/7 activity.

### Inflammatory cytokine response

**Figure 6:**
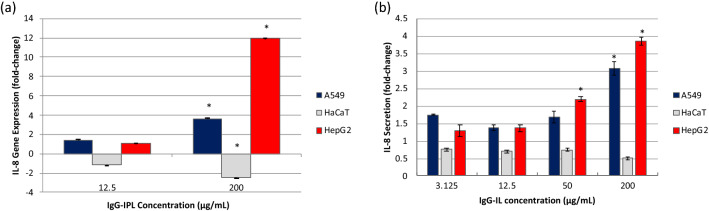
IL-8 (**a**) gene expression and (**b**) cytokine secretion results post-24 h exposure to IgG-IL for A549, HaCaT, and HepG2 cells. Results are presented as fold-change with respect to the no treatment condition. Statistical significance was determined by a One-way ANOVA and a post-hoc Tukey’s Test compared to no treatment, at a confidence of 95% for n = 3, r = 6.

Epitheliod cells produce inflammatory cytokines such as IL-8 in response to xenobiotic stress to initiate immune inflammatory processes^8–10^. Analysis of IL-8 expression revealed a similar concentration dependent effect in both the A549 and HepG2 cells culminating in an approximately twofold increase with the 50 µg/mL treatment and greater than a threefold increase with the 200 µg/mL treatment by 24 h (Fig. 6b). However, the HaCaT cells demonstrated a modestly decreasing trend in IL-8 production with a 49% decrease in IL-8 produced with 200 µg/mL treatment by 24 h (Fig. 6b). The IL-8 effects appear to be regulated at the transcriptional level as qPCR analysis reflect similar trends in IL-8 mRNA expression (Fig. 6a). These results suggest that IgG-IL can induce inflammatory responses in epithelioid cells lines with the most robust responses in the HepG2 cells.

## Discussion

The reactive and unique chemical structure of all the IL formulations make the development of biocompatible IgG-IL a potentially arduous but necessary process that requires high throughput preclinical toxicological analysis to guide development of safe and efficacious products. This study was focused on evaluating the biocompatibility of a representative IgG-IL with multiple human cellular exposure targets in an effort to provide foundational guidance to the preclinical toxicological assessment of subsequent Ig-ILs. Pharmacokinetic evaluation of cationized Ig revealed that it prominently deposited in the liver and lungs, thus guiding our choice of cell lines^5^. In general, the IgG-IL did not cause sustainably significant decreases in cellular viability for all three cell types at concentrations below 12.5 µg/mL. Concentrations at and above 12.5 µg/mL resulted in significant necrotic cell death for A549 and HaCaT cells, while HepG2 treatments demonstrated caspase 3/7 activity suggestive of an apoptotic program of cell death. While, the individual constituents of the IgG-IL, cationized IgG and anion liquid, displayed no significant impact on cellular viability, apoptosis, ROS, or cytokine production.

Elevated ROS levels associated with higher IgG-IL concentrations increased over time and correlated with loss of viability. This profile is suggestive of ROS promoting cell death, however, further studies utilizing anti-oxidants to quench IgG-IL induced ROS will be required to determine a causative relationship between IgG-IL induced ROS and cell death. Furthermore, the concentration dependent IgG-IL-mediated induction of both IL-8 transcripts and protein in A549 and HepG2 cells appears to correlate with the higher-level induction of ROS. Oxidative stress inducing exposures can be associated with an increased IL-8 production at the transcriptional level via the activation of NFκB and AP-1 signaling pathways^11^. Interestingly, HaCaT and HepG2 cells treated with some lower concentrations (< 12.5 µg/mL) exhibited increased ROS levels at later time points suggesting that persistent IgG-IL exposure at low micromolar concentrations could induce oxidative stress that does not correlate with a loss in viability.

The application dependent dosing and pharmacokinetics of therapeutic antibodies can result in a wide range of potential target tissue exposure concentrations. While blood serum concentrations of intravenously administered Ig can be > 100 µg/mL, lower doses or vascular reflection, systemic distribution, and degradation can eventually dilute target tissue (i.e. lung, liver skin) concentrations to low µg/mL and below^7,12–14^. Given that we observed a rough threshold of cytotoxicity between 3.125 and 12.5 µg/mL, an IgG-IL could potentially exhibit either cytotoxicity or biocompatibility based upon the dosing regimen, pharmacokinetics, and Ig structure. A careful understanding of IgG-ILs impact on target cells, including primary sources, will be necessary for the development of a biocompatible IgG-IL product.

This study has established concentration dependent gross cytotoxicity of IgG-IL, but further studies are required to understand the IgG-IL interaction with cells and a subsequent mechanism of cytotoxicity. The cationic modification of the IgG-IL substantially increased the zeta potential of the Ig suggesting an increase in the isoelectric point for the Ig. Hong et al. analyzed the structural activity of hexamethylenediamine cationized Ig and found cationization increased the Ig isoelectric point which potentiates cationic interaction with negatively charged cell membranes and facilitated cellular endocytic transcytosis, and systemic distribution of the Ig^5,15^. While these studies suggest IgG-IL may exhibit enhanced endocytic uptake, the IgG-IL cation is chemically distinct and paired with an anion which could impact cellular uptake. Cellular Ig interaction and uptake can be mediated via Fc receptors that reside on the cell surface and bind and shuttle Ig or Ig/antigen complexes into the cell for antigen degradation (Ig/antigen complexes) or antibody recycling^16^. It is possible that the IgG-IL cation/anion pair could alter FcR binding and recycling functionality. Understanding the role FcRs on IgG-IL cellular interactions could shed light on a mechanism of cytotoxicity and aid in predicting the impact of Ig recycling on the half-life of a therapeutic IgG-IL.

## Materials/methods

### IgG-IL synthesis

An anti-IgG antibody was selected for this study as IgG is a prominent therapeutic isotype that is widely available in bulk quantities necessary for this study^7^. Furthermore, the cell models utilized in this study do not express IgG allowing for the assessment of off target cytotoxicity. For antibody cationization: 1 mg of anti-IgG antibody from rabbit serum (IgG antibodies from rabbit serum (catalog #I5006-50MG), Sigma Aldrich) was dissolved in 1 mL of 0.1 M MES buffer pH 5.0 in a 2 mL microfuge tube and added with 20 µL of 3-dimethylaminopropylamine (Sigma Aldrich) (pH adjusted to 5–6) and 0.25 mg of EDC (1-ethyl-3-(3-dimethylaminopropyl) carbodiimide hydrochloride) from Thermo Fisher. This was incubated for 2 h at room temperature to ensure coupling of 3-dimethylaminopropylamine to antibody. After 2 h, the cationized antibody was dialyzed in 2 L of water from excess coupling reagents using a Slide-A-Lyzer dialysis cassette (3500 MWCO 3 mL volume) (Thermo Fisher) with magnetic stirring at 4 °C over 2 days with 3–4 water changes. 4 mg of poly(ethylene glycol) 4-nonylphenyl 3-sulfopropyl ether potassium salt (Sigma Aldrich) was dissolved in 100 µL of doubly deionized water and added to the cationized antibody to balance charges and form ionic liquid. The cationized antibody/anion pair was lyophilized on a Labconco FreeZone lyophizer for 1 day to remove water and create a protein salt. The protein salt was gently melted at ~ 35 °C to form a viscous antibody ionic liquid. Antibody ionic liquids were characterized by mass spectrometry, zeta potential analysis, and differential thermal analysis (DTA). Mass spectra of unmodified and cationized antibodies were acquired in Reflector Positive Ion Mode on an ABSciex 4800 MALDI-TOF/TOF Instrument. Zeta potential measurements were collected on a Malvern nano-series Zetasizer using a disposable folded capillary cell (Malvern, DTS1070). 0.1 mg of cationized antibody was dissolved in 600 µL of doubly deionized water and placed in a disposable folded capillary cell. The zeta potential of cationized antibody was initially measured to determine overall charge, and then titrated with 0.01 M Poly(ethylene glycol) 4-nonylphenyl 3-sulfopropyl ether potassium salt in water and measured for zeta potential. DTA measurements were performed using a TA Instruments SDT Q 600. The antibody ionic liquid or unmodified antibody powder (~ 2 mg) was placed into a tared alumina crucible with an empty alumina crucible serving as the reference. All data were collected in dynamic mode under flowing argon (100 mL/min) from room temperature up to 100 °C at a rate of 5 °C/min.

### Immuoblot binding assay

For immunoblot, 200 μL of 10 pM, 100 pM, 1 nM, 100 nM, and 1 µM of apoferritin (Sigma) were spotted onto a 4 in. × 0.5 inch PVDF membrane with 0.45 μm pore size (Invitrogen) using a Biorad immunoblotting well plate using previous methods^4^. The entire membrane was then blocked by immersion in ~ 5 mL of tris buffered saline with 0.1% tween 20 (TBST) and 1% bovine serum albumin (BSA) in a sterile petri dish for 2 h. During this time, the membrane was gently agitated on a rocker. After blocking, TBST with 1% BSA was removed and replaced with antibody ionic liquid reconstituted in TBST (20 µg dissolved in 3 mL TBST with 0.1% BSA). The antibody ionic liquid of Anti-horse spleen ferritin or control unmodified antibody was incubated with immobilized apoferritin protein on membrane for 4 h and agitated on a rocker. After 4 h, the reconstituted antibody ionic liquid in TBST with 1% BSA was removed from membrane, and 5 mL of fresh TBST with 1% BSA was added in order to wash excess unbound antibody. The membrane was washed 2 more times with fresh TBST with 1% BSA. After a total of 3 wash steps, a polyclonal human cardiac troponin I Anti-cTnI antibody (H-41, Santa Cruz Biotechnology) was added to membrane at a 1:1000 dilution in 5 mL of TBST with 1% BSA and incubated on a rocker for 12 h. After 12 h, the membrane was thoroughly washed 3 times with 5 mL TBST with 1% BSA. Next, a goat Anti-rabbit secondary antibody conjugated with alkaline phosphatase was added to membrane at 1:1000 dilution in TBST with 1% BSA and incubated for 4 h. After 4 h, the membrane was again repeatedly washed 3 times with 5 mL of TBST and 1 time with double deioinized water. Finally, these binding interactions were detected colorimetrically by exposing membrane to 3 mL of substrate (nitroblue tetrazolium/5-bromo-4-chloro-3-indolyl phosphate) for 5 min. After 5 min, the membranes were extensively washed with double deionized water and a digital image was recorded using a point and shoot 12 MP Canon digital camera, and displayed in Supplementary Figure 1.

### Dispersion

The viscous IgG-IL (IgG modified with 100 equivalent units of DMPDA) was reconstituted at 16.9 mg/mL in RPMI 1640 (Hyclone, GE Healthcare, A549 and HaCaT) or EMEM (Hyclone, GE Healthcare, Eagles’ Modified Essential Media, HepG2) cell culture media supplemented with 10% fetal bovine serum (FBS, ATCC) and 1% penicillin/streptomycin (Pen/Strep, Sigma-Aldrich). Working solutions of 0.25, 0.75, 3.125, 12.5, 50.0, and 200 µg/mL of IL in cell culture media were created for toxicological evaluation.

### In vitro dosimetry

IgG-IL treatment concentrations (0.25, 0.75, 3.125, 12.5, 50.0, and 200 µg/mL) were based on a general µg/mL blood plasma range of therapeutic monoclonal antibodies^7^. These exposures were delivered to each of the different cell lines for 8, 24, or 48 h of aqueous suspension exposure.

### Cell culture

A549 alveolar type-II epithelial cells (ATCC^®^ CCL-185), were selected as an alveolar model for inhalation toxicity, HaCaT kerotinocyte cells for skin exposure and toxicity, and HepG2 hepatocyte (ATCC^®^ HB-8065) cells were utilized to assess hepatotoxicity. The cells were cultured in 96-well or 6-well standard tissue culture plates for toxicity testing or cytokine analysis respectively at a plating density of 40,000 cells per cm^2^. Cell culture medium with or without IgG-IL, IL, or IgG antibody was added to cultures and incubated at 37 °C in a humidified incubator with 5% CO_2_ for the indicated exposure periods.

### Cellular response assessment

#### Cytotoxicity/necrosis (LDH) assessment

Following exposure, 50 µL of conditioned cell culture medium were removed and added to a blank 96-well plate (triplicate measurement per sample). Control cell lysate was utilized as a positive control for LDH leakage. Ten microliters of cell lysis buffer (Promega Corp., USA) were added to each of the lysis control wells and incubated for 30 min. Fifty microliters of cell lysis/conditioned media was added to the 96-well plate. Equal volume of lactate dehydrogenase (LDH) assay substrate (CytoTox-ONE Homogenous Membrane Integrity Assay G7891, Promega Corp., Madison, WI, USA) was added to each of the wells and incubated at 37 °C for 30 min. The fluorescence intensity was then measured with a SpectraMax GeminiXS micro-plate reader at 560 nm excitation and a 590 nm emission wavelengths. Results were plotted as % increase in LDH with respect to the control condition (0 µg/mL treatment concentration).

#### Cell viability

Following removal of conditioned cell culture medium for the LDH assay, a 1:10 AlamarBlue (AlamarBlue cell Viability Reagent DAL1100, ThermoFisher Scientific Inc, Waltham, MA, USA) solution was mixed in warmed cell culture medium and then immediately added to each culture well. Digitonin was utilized as a positive control to disrupt membrane integrity. Cells were incubated for 2 h at 37 °C. Following incubation, fluorescence activity was measured on the spectrometer at 560 nm excitation and 590 nm emission wavelengths. The results were plotted as percent viability with respect to the control condition (0 µg/mL treatment concentration).

#### Apoptosis (Caspase 3/7)

The Caspase-Glo 3/7 Assay is a homogeneous, luminescent assay that measures caspase-3 and -7 activities which are leading indicators of multiple mechanisms of apoptosis, but not conclusive endpoint measurements of apoptosis. In a separate plate from the cell viability/cytotoxicity assays, cells were exposed as described above and at each time point 100 µL of Caspase-Glo 3/7 assay substrate (Caspase-Glo 3/7 apoptosis assay G8093, Promega Corp., Madison, WI, USA) were added to each culture well and incubated at 37 °C for 1 h. In the presence of caspase proteases, the assay substrate is cleaved allowing detection of luciferase luminescence. Luminescence was measured using the luminescence detection setting of the spectrometer. The results were plotted as relative fluorescent units.

#### Reactive oxygen species

Cells were seeded at a density of 15,000 cells/ well in 96-well plates and allowed to adhere for 24 h. Immediately prior to treatment with IgG IPL, 1 µM DCFH-DA (2,7-dichlorofluorescein diacetate, Cell Biolabs OxiSelect Intracellular ROS Assay Kit, San Diego, CA, USA) probe was added to culture wells and incubated for 30 min. Fluorescence emission was read at 538 nm after excitation at 485 nm, according to the manufacturer protocol. Results were plotted as increasing percentage of ROS production with respect to the control condition.

#### IL-8 protein and gene expression

Control and IgG-IL treated cell supernatants were collected after 48 h of treatment. IL-8 (Biolegend) ELISA kits were utilized to quantitatively analyze supernatant IL-8 concentration. The kits were prepared and executed according to the manufacturer protocols. Samples were measured in triplicate averaged together, and data reported as an n = 3 for each condition. IL-8 gene expression experiments were initiated by seeding 250,000 cells/mL in 6-well plates for 24 h before being treated with 0, 12.5, or 200 µg/mL IgG-IPL. After 8 h of treatment, total RNA was collected using the Qiagen RNAeasy kit. Briefly, the cells were lysed using Qiazol. Total RNA was collected via spin column filtration, and purified of DNA via RNase free DNase. RNA was quantitated by spectrometry. Reverse transcription was performed using 1 µg of RNA mixed with high-capacity cDNA Reverse Transcription kit master mix (ThermoFisher Scientific, Inc.), and transcribed into cDNA utilizing manufacturer protocol. Real-time quantitative PCR analysis was conducted using Taqman Gene Expression assays for and IL-8 (Hs00174103_m1). Equal amounts (10 ng) of cDNA were added to each of the wells and mixed with TaqMan Universal Master Mix II (no UNG, Applied Biosystems Inc.), the TaqMan gene expression assay probes, and DNase-RNase-free water. HPRT-1 (Hs02800695_m1, TaqMan gene expression assay) was used as an internal reference control. Real-time quantitative PCR was conducted utilizing the Taqman assay protocol with a Stratagene MX3000P thermocycler, and relative quantification was calculated using MxPro Mx3005P v4.10 software (Stratagene, Agilent Technologies, Santa Clara, CA: https://www.agilent.com/en/product/real-time-pcr-(qpcr)/real-time-pcr-(qpcr)-instruments/mx3000-mx3005p-real-time-pcr-system-software/mxpro-qpcr-software-232751) via the delta-delta cycle threshold method (2^(−ΔΔCt)^).

### Statistical analyses

Each condition was represented with eight independent replicates, analysis was conducted in triplicate measurement, and the mean value and standard error of the mean plotted. Two-way ANOVA statistical analysis was conducted in Microsoft Excel, and post-hoc Tukey’s comparison analysis of means was performed using Graphpad Prism 8 to determine statistical differences across condition variables.

## Supplementary Information

Supplementary Information.
