# Supplementary material for: Examining cellular responses to reconstituted antibody protein liquids
Source: Sci Rep. 2021 Aug 23;11:17066. doi: 10.1038/s41598-021-96375-8 (PMC8382709; doi:10.1038/s41598-021-96375-8)
Supplement: Supplementary file 1 — Supplementary Information. [file 41598_2021_96375_MOESM1_ESM.docx]

**Examining Cellular Responses to Reconstituted Antibody Protein Liquids**

M. Tyler Nelson ^1^, Joseph M. Slocik ^2,3^, Eric J. Romer ^1,3^, Cassandra I. Mankus ^4^, Richard T. Agans ^5^, Rajesh R. Naik ^1^, Saber M. Hussain ^1^

^1^ 711^th^ Human Performance Wing, Airman Systems Directorate, Molecular Mechanisms, Air Force Research Laboratory, Wright-Patterson Air Force Base, Ohio 45433

^2^ Materials and Manufacturing Directorate, Air Force Research Laboratory, Wright-Patterson Air Force Base, Ohio 45433

^3^ UES Inc., Dayton, Ohio 45433

^4^ Oak Ridge Institute for Science and Education, Oak Ridge, Tennessee

^5^ The Henry M. Jackson Foundation, Bethesda, Maryland 20817

Corresponding author: M. Tyler Nelson, [nelsonmt05@gmail.com](mailto:nelsonmt05@gmail.com)

**Supplementary Information**

**Supplementary Figure** 1

Supplementary Figure 1: Immunoblot binding assay of Anti-horse spleen ferritin in the form of native antibodies (Top) and antibody ionic liquids (Bottom) at room temperature. Apoferritin is immobilized on nitrocellulose membrane. (Note: images taken from native gels without excision)

Supplementary Figure 2


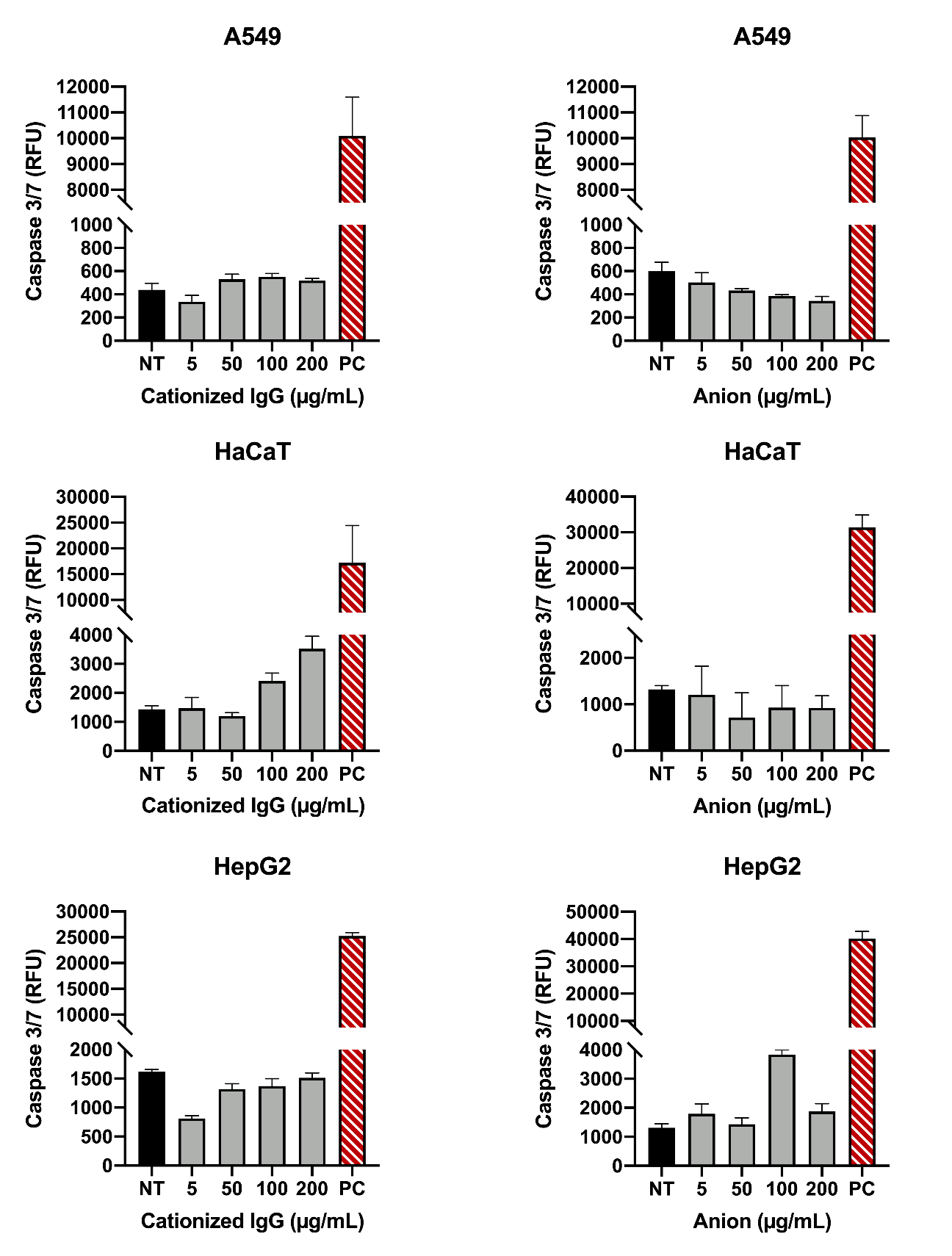


Supplementary Figure 2: Caspase 3/7 luminescence results for A549, HaCaT, and HepG2 cells exposed to cationized IgG or anion liquid for 24 hours. The positive control (PC), was a spike in of 1 µM staurosporine (Promega, Inc.) for 4 hours prior to reading the plates for luminescence. No statistically significant results were indicated. * denotes statistical significance, n = 4, p < 0.05, One-way ANOVA, Dunnett’s Post-Hoc test for statistical difference

Supplementary Figure 3


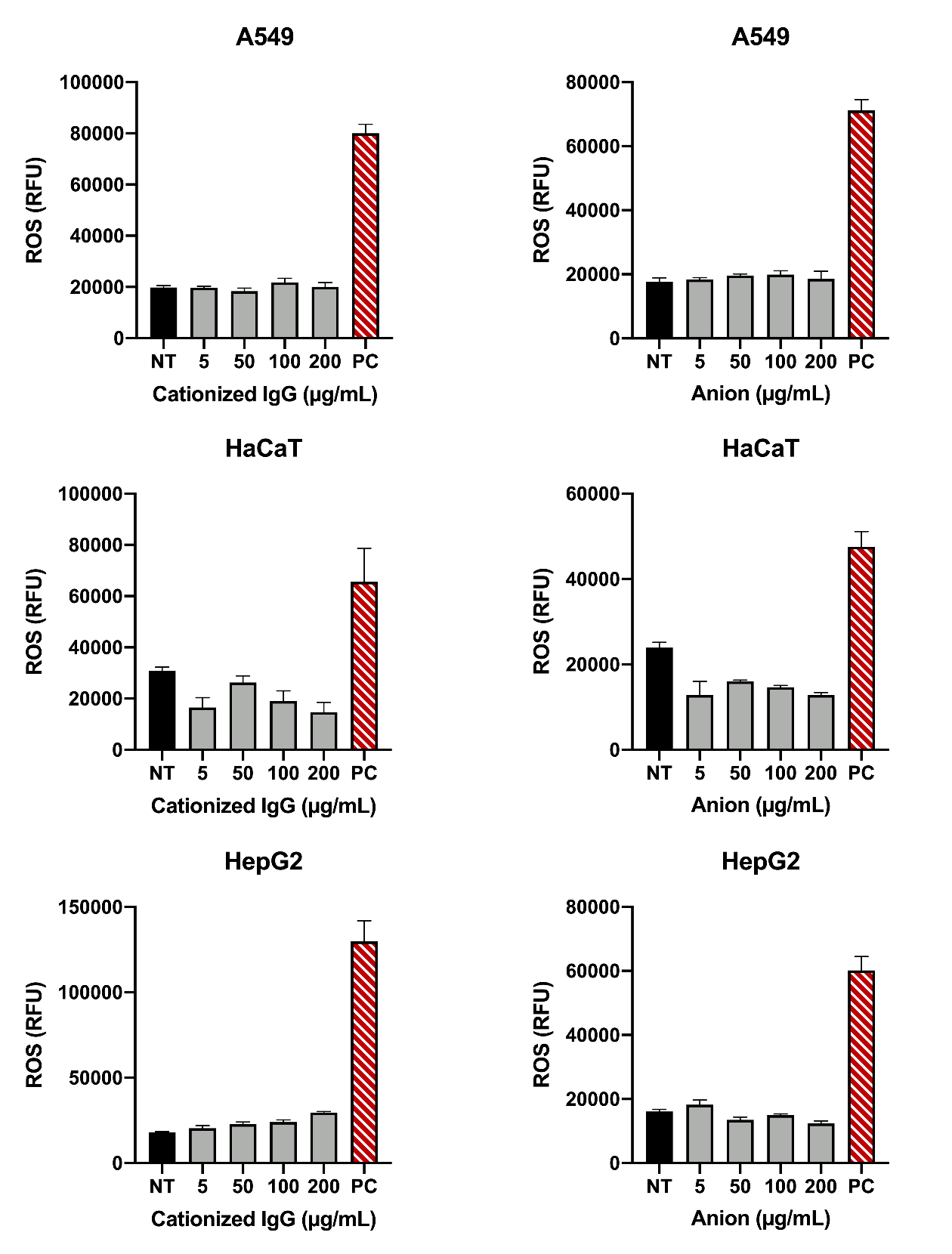


Supplementary Figure 3: Reactive oxygen species (ROS) fluorescent probe results for A549, HaCaT, and HepG2 cells exposed to cationized IgG or anion liquid for 24 hours. The positive control (PC), was an injection of 100 µL of 100 µM hydrogen peroxide (30% v/v analytical grade, Sigma-Aldrich) for 30 minutes prior to screening the cell culture plates on the spectrophotometer. No statistically significant results were indicated. * denotes statistical significance, n = 4, p < 0.05, One-way ANOVA, Dunnett’s Post-Hoc test for statistical difference


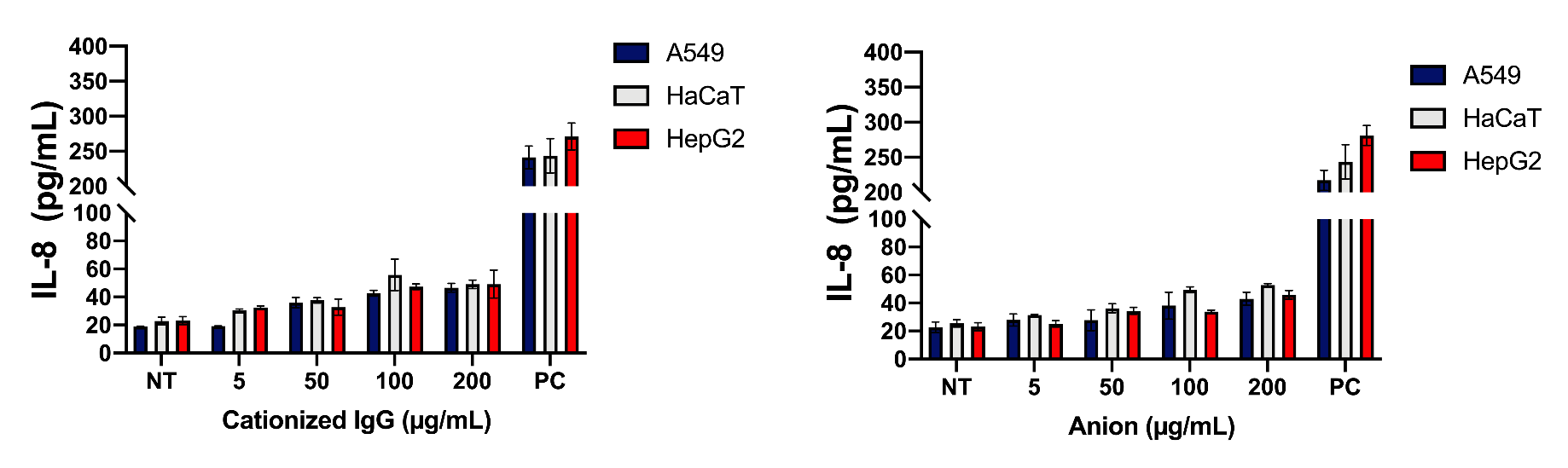
Supplementary Figure 4

Supplementary Figure 4: IL-8 ELISA results for A549, HaCaT, and HepG2 cells exposed to cationized IgG or anion liquid for 24 hours. The positive control (PC) was a treatment of 0.1 µM staurosporine (Promega Inc.) to induce cell stress over the 24-hour exposure period. No statistically significant results were indicated. * denotes statistical significance, n = 4, p < 0.05, One-way ANOVA, Dunnett’s Post-Hoc test for statistical difference
